# Exploring influencing factors in breast cancer survivors’ experience in Lebanon

**DOI:** 10.3389/fpsyg.2022.965825

**Published:** 2022-08-23

**Authors:** Marwa Saab, Xue Han

**Affiliations:** School of Psychology, Northeast Normal University, Changchun, China

**Keywords:** cancer survivor, breast, family support, religious beliefs, body image, children, Lebanon

## Abstract

**Background:**

The research objective was to investigate social and cultural factors affecting breast cancer survivors’ experiences in Lebanese.

**Methods:**

A snowball sampling of 20 breast cancer survivors participated in the study. Semi-structured open-ended interviews were used to collect data.

**Results:**

The results showed that family support and religious beliefs were the primary supporting sources for breast cancer survivors. On the other hand, their body image and children were the major concerns. Thus, family and religious beliefs were needed to overcome breast cancer’s daily burden.

**Conclusion:**

Women with breast cancer perceived their cancer experience through their social roles, reflecting a concern for image and role preservation.

## Introduction

Breast cancer (BC) represents one of the highest incident rates of cancers worldwide. In Lebanon, BC was accounted for 37.9% or 6,550 of the total new cancer cases in women in 2018, and 18.6% or 3,216 of total cancer cases in both sexes, representing the highest incident rates of cancer in the country (WHO, 2020). BC may cause body deformation that alters social body images as well as jeopardizes individuals’ social roles and activities (Zimmerman, 2004). “(BC) is an instigator of many fears with patients, primarily the deepest existential fear, the fear of death, separation and isolation from their loved ones, as well as a fear of deterioration and pain” (Michael et al., 2000, p. 343).

Previous studies that focused on post-treatment psychological states of breast cancer survivors (BCS) found that family and emotional support are necessary during BC treatment (Cvetković and Nenadović, 2016). Also, previous research highlighted the importance of emotional and practical support from the family, doctors, medical staff, and support groups (Ashing-Giwa et al., 2004; Thewes et al., 2004; Snyder and Pearse, 2010; Knobf, 2011). In addition, cultural and health beliefs played a role in BCS in terms of seeking treatment or coping with cancer. However, family support can represent either help or stress depending on the family’s expectations for BCS’s roles and reactions toward the disease (Meacham et al., 2016; Hubbeling et al., 2018). For example, studies have found that BCS expressed feelings of sadness, embarrassment, decreased self-worth, and attractiveness (Ashing-Giwa et al., 2004; Hu et al., 2021). Alternatively, felt embarrassed or anxious due to their body shape (Bloom et al., 2004). However, other research found that family and spouse helped BCS positively adapt to body image change (Hubbeling et al., 2018).

Similarly, some research found that an increase in the level of social support during the BC period may lead to a longer-term posttraumatic growth compared with the usual level of social support (Romeo et al., 2019). Likewise, BCS with a high level of family avoidance were reported to have more mental health problems than those with low (or none) family avoidance (Mallinger et al., 2006). Also, other research resulted that avoidant coping mediates the relationship between well-being and distress in BCS (Cohee et al., 2021). Moreover, studies that investigated the role of a spouse or intimate partner in BCS post-treatment period found that the BCS reported that spouse or intimate partner has been providing all forms of social support (Muhamad et al., 2011), and the most important confidence (Figueiredo et al., 2004). Intimate partner support was also more influential over the long term (Shrout et al., 2021). Women with supportive spouses practiced more regular activities (Mackenzie, 2015). However, BCS with partners who needed support and information during the post-treatment period reported higher depression and anxiety levels and used more emotion-oriented coping strategies than their counterparts (Pauwels et al., 2012). Research on dyadic coping resulted that it may improve relationship functioning between cancer patients and their partners (Traa et al., 2015). Common dyadic coping was related to lower psychological distress in cancer patients and their partners (Meier et al., 2019). Similarly, dyadic personal growth developed sooner in female partners of cancer male patients (Künzler et al., 2014).

Moreover, in general, previous research with BCS from the United States (Ashing-Giwa et al., 2004; Bloom et al., 2004) expressed high spiritual beliefs in God’s will in terms of living with cancer or in treatment, which was considered as the central pillar for survival. Similar results were presented in two meta-analyses, one by Knobf (2011) on the psychological response of BCS and another by Park (2013) on spirituality and meaning-making. A recent study by Nilsen et al. (2021) resulted that the BC experience led to a shift in meaning in life. BCS approached and understood life differently after having BC, which was one coping tool. Hu et al. (2021) resulted that one of the coping strategies BCS had been the support from their communities or individuals external from the family.

Furthermore, BCS had higher levels of depression and anxiety 5–6 years after diagnosis compared to 40 weeks after diagnosis. Age (older BCS) and having comorbidities were associated with depression and anxiety, while children were associated with depression (Breidenbach et al., 2022). On the other hand, Carreira et al. (2021) found that younger BCS were associated with higher depression and anxiety. Moreover, BCS compared with non-BC had lower quality of life.

A recent study on defense mechanism functioning in BC inpatients in Lebanon found that married BC inpatients displayed a higher defense mechanism than unmarried BC inpatients. In addition, the study found that affiliation is one of the most frequent defense mechanisms displayed by BC inpatients, reflecting a tendency to reach out to family members and the surrounding environment (Saab et al., 2021).

Thus, based on previous research and the high incidence rates of BC in Lebanon, this study aimed to investigate what social and cultural factors affect BCS’ experiences with the disease in Lebanese society. Previous research had somehow contradicting results, reflecting similarities and differences across cultures or societies. In this study, the focus is to explore what factors may influence BCS in the Lebanese society, which had not been studied before.

## Materials and methods

### Participants

Due to patient’ confidentiality reasons, BCS support groups refused to share their members’ information. Therefore, we used snowball sampling. Twenty BCS women (age *M* = 51, range: 24–74) were recruited. Participants were eligible if they: (1) lived in Lebanon before having cancer or are Lebanese; (2) able to communicate in Arabic fluently; (3) completed cancer treatment; (4) not currently under any cancer treatment; (5) did not have any mental or psychological disease that will affect the results of this research; and (6) are 18 years old or above.

Fifteen of the participants were married at the time of data collection, four were single, and one was widowed; among the married, 70% have children. The majority of participants were unemployed or retired (70%). In addition, 30% of participants had no insurance. Regarding breast cancer stages, 70% of participants were initially diagnosed as stage II, while the rest were stage I and III (10% and 20%, respectively). Moreover, the period since participants completed their cancer treatment and was considered cancer-free by their oncologists ranged between 4 months and 15 years. Concerning treatment, 90% of participants did chemotherapy, 70% did radiotherapy, 40% underwent a mastectomy, and 15% did breast reconstruction.

### Design

This is a qualitative study. Semi-structured interviews were conducted with BCS among the Lebanese society, see Table 1 for details.

**Table 1:** Interview questions.

Main questions	Please tell me about your experience in BC
	Please tell me about whether someone was with you during this (or these) periods
	Please tell me what are your usual daily activities
Some follow-up questions^a^	What changed during this period?
	What changed after this period?
	Was there anything that helped you during this process?

a Please note that these questions are a follow-up of a previous answer.

### Procedure

The data were collected through a snowballing technique. First, some participants were asked by their oncologist or the hospitals during their yearly follow-up procedure if they would like to participate in this study. If participants agreed to participate in this study, they were asked to recommend (if possible and under consent) other participants.

Semi-structured open-ended interviews were conducted in a place appointed by the participants to ensure their comfort and privacy. Interviews were designed to understand participants’ experience of BC. The interview questions were constructed based on previous research findings that BCS expressed a support or burden system during-and post-treatment (e.g., Ashing-Giwa et al., 2004; Thewes et al., 2004; Snyder and Pearse, 2010; Knobf, 2011; Cvetković and Nenadović, 2016; Romeo et al., 2019). Therefore, three main questions were constructed to be asked to all participants: (1) “Please tell me about your experience in BC,” (2) “Please tell me about whether someone was with you during this (or these) periods,” and (3) “Please tell me what are your usual daily activities.”. Each of these questions was followed by further questions related to the BC experience and depending on the flow that each participant set (please see Table 1 for some of the follow-up questions). Interviews were audio-taped, and participants were told twice, before and after data collection, of the anonymous of their answers. Participants could also talk to the researcher in case they had any concerns. In addition, because several participants were concerned about sharing their personal life with strangers, they were assured that the full interviews from this research would only be visible to the researchers and that they could withdraw from this research at any moment prior to its publication. Following this, participants signed informed consent. The ethical committees approved this study from Northeast Normal University School of Psychology (2017005) and under the 1964 Helsinki Declaration and its later amendments.

Data collection was terminated after the last three interviews as they did not add any new information that was not mentioned by other participants (Ritchie et al., 2013).

### Data analysis

**Figure 1:**
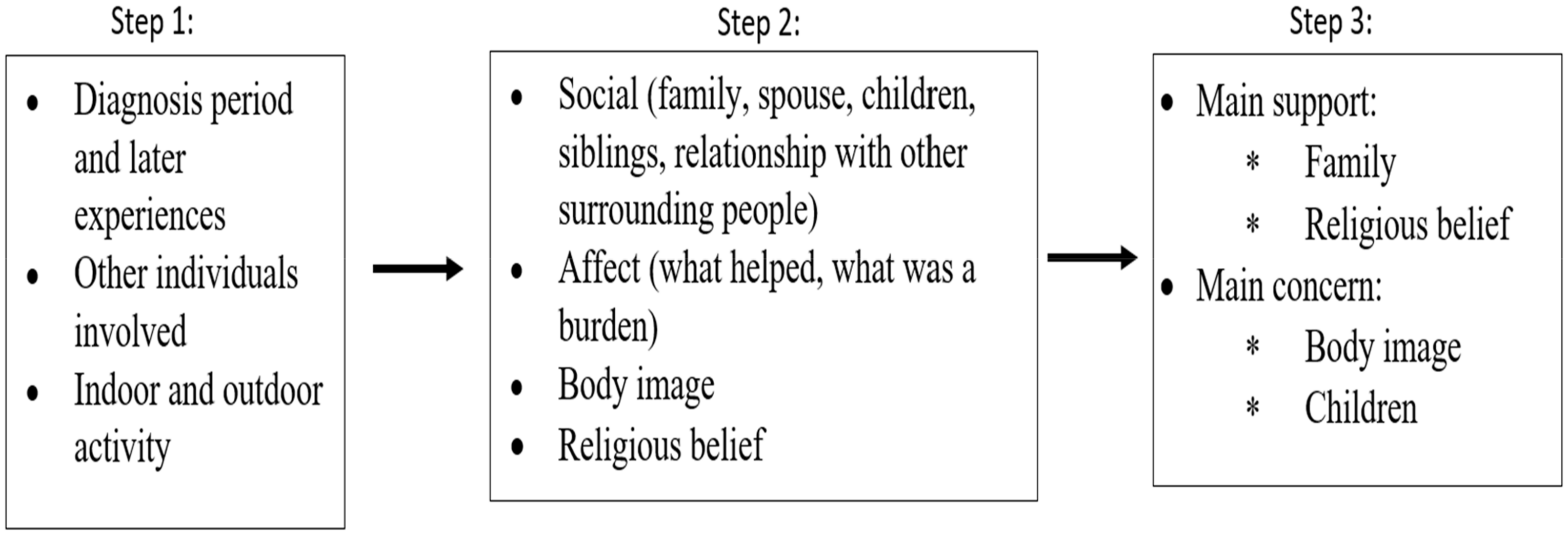
Thematic coding procedure.

Thematic coding was used in data analyses, the six-phase steps suggested by Braun and Clarke (2006) were used. Following the six-phase thematic coding, the researchers were first getting familiar with the data. In phase two, three initial codes were formed, which reflect the three main questions asked in the interviews. These initial codes led to phase three, which is searching for themes. Four themes were generated in phase three. In the next phase, a fellow researcher familiar with the study reviewed these themes. Then, phase five was agreed upon with the researchers, which is defining the themes. The four themes were then rendered into two larger themes. Finally, the researchers proceeded to phase six, producing the report. Figure 1 summarizes the coding procedure.

## Results

The thematic coding resulted in two main themes: (1) main supports that include family support and religious belief, and (2) major concerns that include body image and children. BCS expressed two main supporting sources, family support and religious belief, in which both of these supporting sources were mentioned at least twice by each participant. Family support included support from parents, siblings, husband, and children. Participants had at least one of these family members as their main supporters. Moreover, religious belief was abundantly expressed. The focus was not on social expressions usually used as “in God’s will” but on phrases reflecting the participants’ religious perception. Participants also experienced the BC period through their perception of themselves as women and their social role, which was a major concern. Participants’ main concern was their body image, reflecting them as women, which was expressed at least twice by each participant throughout the interviews. In addition, participants who are mothers had their children as another concern they had to deal with during their BC period.

### Main support

#### Family

Overall, family support positively impacted BCS; however, it differed between married and single participants. Family support also differed at the level of the specific supporter. The majority of married participants (11) had their primary source of support from the husband, while single participants had their primary source of support from their parents and/or siblings. Some participants had family support from one source of the family but not the other, such as having support from husband and parents but not from siblings. Moreover, participants did not include any household duties or taking care of the children when mentioning the husband as supportive. The husband’s support was bound to financial, physical – as being there with the participants, and emotional – as body image enhancement. These examples illustrated family support:

“My husband was my main supporter. He used to say; I do not care how much the doctor's fee is, when you want to go out or somewhere, and you cannot drive, do not disturb anyone take a taxi. My mother was beside me, but she used to worry and complain a lot (speaking about the sickness) … My sisters were affected for sure, but at the same time, they started to be more afraid of what might happen to them. They did not want to listen; they did not want to know; they became obsessed. They started visiting doctors on the slightest things that happened to them. They did not want to speak to me or to stay with me; they do not want to know anything about this problem.”

“What helped most was the presence of my family beside me, and my husband supported me a lot. For example, if he finds me upset, he will tell me, what is the maximum that will happen? You will get better, and we will live our lives; my mom was still alive. She had always been with me … Once when I was still in the hospital, I was taking a shower, and my hair started falling; I do not know how to explain this feeling, it bothered me a lot, I started crying. My husband said I would not let you see yourself without hair. He used to tell me, now, you are looking better than before the treatment like they used these words to convince me … He was very supportive.”

#### Religious belief

Participants strongly associated religious belief with acceptance of breast cancer, its treatment process, and possible mortality outcome. The majority of BCS in our study expressed the control of God on their life and death using expressions such as “this is something from our God,” “it is fate,” “I put my life in the hands of God,” and “what comes from God is good.” Some BCS saw cancer as “a sign from God,” or a punishment as one said that: “I used to cry daily, I felt that God punished me.” Religious acts were also a means of releasing stress from BC, such as praying and increased belief. Two participants put on the Islamic hijab after having cancer; however, one participant removed it when her hair grew back. These two participants did not link putting on the hijab with having cancer.

### Major concerns

The results showed body image as the main concern in all participants. Children raising and their future were another main concern for mother participants with children still needing nurturing.

#### Body image

Participants demonstrated body image as the most challenging part of BC. Even though not all participants did chemotherapy, which will cause hair loss and nail deformation, the majority had problems with their body image change. Participants’ main concern was the external figure, such as hair and breast shape. Some participants covered their heads with a headscarf or the Islamic hijab. In addition, many participants changed their daily routine to avoid being seen without a headcover. Hence, participants used make-up as a solution. Throughout the interviews, they emphasized the link between being a woman and not losing your figure. However, even with make-up, some participants could not bear to look at themselves in the mirror.

#### Children

Six of the participants have children who still need nurturing. These participants expressed worry and fear about their children’s future and well-being during treatment and, in case, after they passed away. In addition, participants expressed willingness to survive BC to raise their children. This example illustrated this as the following:

I used to look to my daughter and think how can she continue without me, because she is a kid of 12 years old, and we do not have anyone … One day I decided that for her, I should continue, I should live in a normal way, I should be happy, and she should not feel anything. For this same reason, that really upset me helped me a lot because I put in front of me one goal that I will live happy with this kid, and this is what made me strong.

## Discussion

This study is the first to explore the social and cultural factors influencing BCS experience with the disease in Lebanon. In general, our results showed that the most influential social and cultural factors for BCS are family support, religious belief, body image, and children. BCS dealt with BC through their personal beliefs of women’s social roles and motherhood. This is reflected in the results in BCS’s supporting sources as family and religious beliefs and concerns about body image and children.

The family presented a supportive factor for BCS, which is consistent with previous research (Meacham et al., 2016; Hubbeling et al., 2018). Saab et al. (2021) also found that family affiliation played a role in BC inpatients during cancer treatment. Moreover, the husband’s support also aligned with previous research (Figueiredo et al., 2004; Muhamad et al., 2011). However, the husband’s support was emotional, physical, or financial, yet, it did not include children nurturing or any unpaid job representing household activities. Hence, the husband’s support was perceived from participants’ roles as women, wives, and mothers. Taking care of children, cleaning the house, or cooking were not present as a supporting element that the husband did or could do. Mackenzie (2015) also showed that although BCS participants highlighted the husband as influential, the support was according to participants’ belief in motherhood, unpaid and paid jobs, and social roles.

Another supportive factor was religious belief. Many BCS needed spiritual sublimation to overcome the fear of death and be able to go back to their daily routine. The results were also found in previous research (Ashing-Giwa et al., 2004; Bloom et al., 2004; Knobf, 2011).

Furthermore, the results showed two major concerns. BCS who have children who still need nurturing perceived their children’s lives without them as their major concern. Mother participants perceived themselves as the primary caregiver for their children regardless of whether they had paid jobs or not. The result reflected that taking care of the household and children is a women’s social role.

Moreover, body image represented another aspect of how BCS perceived themselves as women with specific roles and appearances. The highlighted concern was hair loss, which could be due to our participants directly relating hair to the physical appearance, since the breast can be covered and not shown to the external world. Women with BC could not identify as having a lesser physical appearance than what they are supposed to have. Participants focused on their image through people’s eyes. However, this form of projection is also related to their image and values. Thus, women in Lebanese society consider body image to be the main representative of themselves.

Although we explored and discovered some of the possible influencing factors in BCS, this study still has some limitations. First, it was not a longitudinal study, in which it could compare people before and after having BC. Second, it also did not compare BCS with non-cancer individuals. Third, due to the absence of a detailed national data registry about BCS, survival rate, or distribution of BC patients, we were only be able to use the snowball sampling technique to collect data, which may not be representative. Fourth, we did not control for BCS’s survival years when recruiting participants, which may also be important. Moreover, although our focus was only on the BCS, not their family members or treatment, these could be very useful for designing intervention programs. For example, some studies showed that an intervention involving the direct caregiver, a family member, or the spouse could help both BCS and their families cope during this period and in the long run (Bellin et al., 2015; Hsiao et al., 2016). Also, previous research that focused on dyadic coping of cancer patients and their partners highlighted the need for the partner involvement in the BC patients and BCS treatment and post-treatment period (Künzler et al., 2014; Traa et al., 2015; Meier et al., 2019). Therefore, future studies could focus on group therapy involving direct family members or a broader social surrounding.

In conclusion, this is an exploratory study, since it is the first one to investigate influencing factors in BCS in Lebanon. It can form a base for the understanding of BCS experience and shine the light on future research.

## Conclusion

BCS experienced two supports and two concerns as the result of the disease. Family and religious beliefs played a supportive role in overcoming BC’s daily burden, while body image and child nurturing presented as major concerns. Generally, BCS perceived their cancer experience through their social roles, reflecting a concern of image and role preservation.

## Data availability statement

The datasets presented in this article are not readily available because “The data are not publicly available due to privacy or ethical restrictions.” Requests to access the datasets should be directed to marwa_saab@hotmail.com.

## Ethics statement

The studies involving human participants were reviewed and approved by Northeast Normal University School of Psychology (2017005). The patients/participants provided their written informed consent to participate in this study.

## Author contributions

MS and XH contributed to the conception and design of the study, manuscript revision, and read and approved the submitted version. MS collected the data, contributed to the procedure and analysis. All authors contributed to the article and approved the submitted version.

## Funding

This research was funded by grant 20210101009JC from the Jilin Provincial Department of Science and Technology, awarded to XH.

## Conflict of interest

The authors declare that the research was conducted in the absence of any commercial or financial relationships that could be construed as a potential conflict of interest.

## Publisher’s note

All claims expressed in this article are solely those of the authors and do not necessarily represent those of their affiliated organizations, or those of the publisher, the editors and the reviewers. Any product that may be evaluated in this article, or claim that may be made by its manufacturer, is not guaranteed or endorsed by the publisher.
